# A Systematic Approach to Review of *in vitro* Methods in Brain Tumour Research (SAToRI-BTR): Development of a Preliminary Checklist for Evaluating Quality and Human Relevance

**DOI:** 10.3389/fbioe.2020.00936

**Published:** 2020-08-07

**Authors:** Mike Bracher, Geoffrey J. Pilkington, C. Oliver Hanemann, Karen Pilkington

**Affiliations:** ^1^School of Health Sciences, Faculty of Environmental and Life Sciences, University of Southampton, Southampton, United Kingdom; ^2^School of Pharmacy and Biomedical Sciences, University of Portsmouth, Portsmouth, United Kingdom; ^3^Institute of Translational and Stratified Medicine, University of Plymouth, Plymouth, United Kingdom; ^4^School of Health and Social Care Professions, University of Portsmouth, Portsmouth, United Kingdom

**Keywords:** *in vitro*, quality appraisal, evaluation, critical appraisal, brain tumour, cancer, systematic review

## Abstract

**Background:**

A wide range of human *in vitro* methods have been developed and there is considerable interest in the potential of these studies to address questions related to clinical (human) use of drugs, and the pathobiology of tumours. This requires agreement on how to assess the strength of evidence available (i.e., quality and quantity) and the human-relevance of such studies. The SAToRI-BTR (Systematic Approach To Review of *in vitro* methods in Brain Tumour Research) project seeks to identify relevant appraisal criteria to aid planning and/or evaluation of brain tumour studies using *in vitro* methods.

**Objectives:**

To identify criteria for evaluation of quality and human relevance of *in vitro* brain tumour studies; to assess the general acceptability of such criteria to senior scientists working within the field.

**Methods:**

Stage one involved identification of potential criteria for evaluation of *in vitro* studies through: (1) an international survey of brain tumour researchers; (2) interviews with scientists, clinicians, regulators, and journal editors; (3) analysis of relevant reports, documents, and published studies. Through content analysis of findings, an initial list of criteria for quality appraisal of *in vitro* studies of brain tumours was developed. Stage two involved review of the criteria by an expert panel (Delphi process).

**Results:**

Results of stage one indicated that methods for and quality of review of *in vitro* studies are highly variable, and that improved reporting standards are needed. 129 preliminary criteria were identified; duplicate and highly context-specific items were removed, resulting in 48 criteria for review by the expert (Delphi) panel. 37 criteria reached agreement, resulting in a provisional checklist for appraisal of *in vitro* studies in brain tumour research.

**Conclusion:**

Through a systematic process of collating assessment criteria and subjecting these to expert review, SAToRI-BTR has resulted in preliminary guidance for appraisal of *in vitro* brain tumour studies. Further development of this guidance, including investigating strategies for adaptation and dissemination across different sub-fields of brain tumour research, as well as the wider *in vitro* field, is planned.

## Introduction

There is currently a drive to review the use of animals in research for both scientific and ethical reasons. A wide range of *in vitro* methods have been developed and, increasingly, there are suggestions that these can replace the use of animals in research (NC3Rs, 2020). In order for *in vitro* studies to be considered for replacement of *in vivo* (animal) studies to answer questions related to the clinical (human) use of drugs and pathobiology of tumours, there must be agreement on the strength of evidence available (i.e., the quality and quantity of studies) as well as their relevance. Judging the strength of evidence requires that all relevant research is located, each research study is assessed for quality and, if appropriate, the results of the individual research studies are combined to give an overall ‘answer’ and/or a clear picture of the current research on the topic in question. This process can also reveal poor research practises, unreliable reporting of research and unnecessary replication and duplication (Hartung et al., 2019). Any such practises, if left undetected, would render efforts to replace animal research less likely to gain acceptance.

Methods for assessing clinical (human) studies are well-developed, led by organisations such as the international Cochrane collaboration (Higgins and Green, 2011). ‘Systematic reviews’ of the evidence are regularly published (over 140,000 systematic reviews are listed on PubMed as of March 2020). Well-conducted systematic reviews of clinical studies are widely used as the basis for clinical decisions.

A parallel development has taken place for animal studies. CAMARADES (Collaborative Approach to Meta-Analysis and Review of Animal Data from Experimental Studies) is an initiative to improve the design, conduct, analysis and reporting of animal experiments (CAMARADES, 2020). By means of ‘precise and robust’ overviews of existing data through systematic review and meta-analysis, CAMARADES aims to clearly demonstrate where further experiments are necessary, avoiding unnecessary replication. The CAMARADES initiative has generated interest and collaborative efforts on a global scale with five national coordinating centres. This is seen as crucial in efforts to reduce animal experimentation.

Reduction strategies, however, constitute only one of the Three Rs (Refinement, Reduction, and Replacement) – the underlying principles of ethical and humane use of animals in research (NC3Rs, 2020). The third principle, replacement, as described above, requires that a desired scientific goal is achieved by approaches other than those involving live animals, such as through use of *in vitro* studies. As with the CAMARADES initiative ‘precise and robust’ overviews of existing research are essential to provide a clear picture of the research (Nuffield Council on BioEthics, 2005). However, as Hartung et al. observe, ‘[while] [m]any areas have developed reporting standards and checklists to support the adequate reporting of scientific efforts… *in vitro* research still has no generally accepted criteria… [and] such a culture may undermine trust in the reproducibility of animal-free methods’ (Hartung et al., 2019). Thus, there is a need to evaluate and develop current practises for assessing quantity and quality of *in vitro* studies of brain tumours and their potential to replace *in vivo* (animal) studies.

As Hartung et al. (2019) indicate, issues in reporting are not restricted to specific areas of interest (such as brain tumours) but encompass the broad field of *in vitro* research. Searches on a major scientific database (PubMed) reveal that while reviews have been published and described as systematic reviews of *in vitro* studies, many fail to apply key principles and processes expected of such studies. For example, one publication reported the databases searched and inclusion criteria, but not whether any quality criteria were applied (Laaksonen et al., 2010). A second ‘systematic review’ assessed each study based on two criteria defined by the authors (type of publication and whether there was a ‘comparable baseline’), and reported the lack of generally accepted evaluation criteria for *in vitro* studies (Xiao et al., 2011). A third review revised an existing tool for assessing diagnostic studies using four selected criteria (Deng et al., 2016). Few details are reported on exactly how these criteria were applied. Another study attempted to provide an overview of guidance systems with evaluation criteria for *in vitro* studies on chemical toxicity (Lynch et al., 2016). The criteria compared were from four sources [Animal Research: Reporting of *in vivo* Experiments (ARRIVE) (Kilkenny et al., 2010), Klimisch et al. (1997) on evaluating the quality of toxicological data; OECD Guidance Document on the Validation and International Acceptance of New or Updated Test Methods for Hazard Assessment (OECD, 2005); Toxicological Data Reliability Assessment Tool (ToxRTool) (Schneider et al., 2009)]. The criteria include reporting requirements, categories to be scored and items to be assessed. Few criteria were common to all 4 sources. Furthermore, while criteria for assessment of the quality of studies is crucial for unbiased, reliable reviews of the research literature, assessment of relevance of the technique or method employed is also a key element.

Both development of reporting standards for *in vitro* research (Hartung et al., 2019) as well as adoption of existing guidance (Pamies et al., 2017; Hartung et al., 2019) remain issues across the broad field of *in vitro* research. This study specifically focuses on brain tumour research, with the intention of providing a model for other areas. We have selected brain tumour as a particular area for study because although many brain tumours can be cultured in the laboratory with relative ease, there are specific challenges in gaining accurate biological information from cells which have been removed from such a complex multicellular organ as the brain. Not only are brain tumour cells reliant on the non-neoplastic cells such as glial and immune cells for their resistance to therapeutics, but they are also reliant on the very special vasculature of the brain and indeed the blood brain barrier which protects against toxins but inhibits delivery of therapeutics. Provision of sophisticated, complex 3D models of the brain and its vasculature, including organoids, induced pluripotent stem cells and blood brain barrier elements for pre-clinical drug delivery and sensitivity are perhaps the most complex forms of all human tissue *in vitro* systems and, if we can produce best practise criteria for this area we can roll this out for many other areas of research.

The overall aim of the SAToRI-BTR project is to explore how *in vitro* studies could be presented as a body of knowledge in the form of a rigorous and comprehensive systematic review, to assess the potential for replacement of animal studies for answering specific questions in brain tumour research. SAToRI-BTR seeks to address these challenges by assessing reviews of existing studies (published systematic reviews) and identifying areas for potential improvement and investigating current practise and views on how *in vitro* studies of brain tumours should be assessed, leading to agreed criteria.

The aim of the study reported in this paper was to explore potential methods for the systematic identification, and assessment of quality and appropriate use of *in vitro* studies through a process involving identification of existing criteria which were subject to expert review in order to develop draft criteria.

## Materials and Methods

**FIGURE 1:**
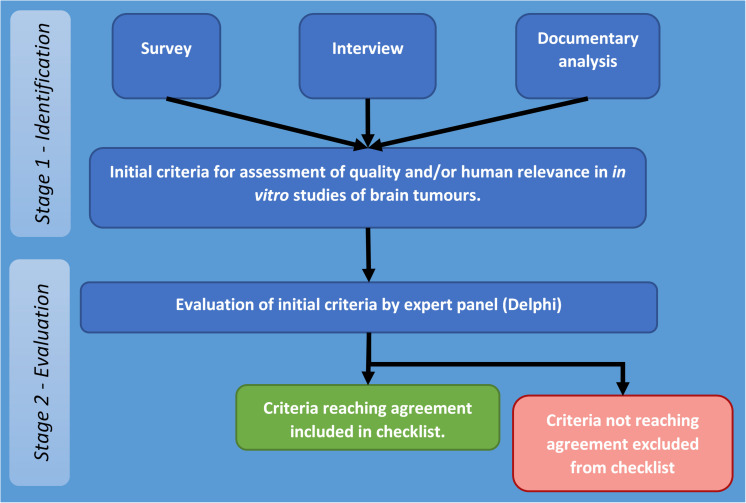
Flow diagram of stages and procedures in identification and evaluation of criteria for assessment of quality and/or human relevance in *in vitro* studies of brain tumours.

The project to develop a set of appropriate criteria for assessment of quality and human relevance in *in vitro* studies of brain tumours was carried out in two stages. The first stage involved identification for potentially relevant criteria through collection and analysis of appropriate data (stage one), and the second stage focused on obtaining agreement on identified criteria by an expert panel by means of a Delphi process (stage two – see Figure 1).

The overall process followed that developed by the EQUATOR (Enhancing the QUAlity and Transparency Of health Research) Network which was used for development and agreement on reporting guidelines for systematic reviews (PRISMA: Preferred Reporting Items for Systematic Reviews and Meta-Analyses) (Moher et al., 2009). This required documenting the need for a set of guidelines by reviewing previously published systematic reviews and the methods and reporting of these, reviewing existing literature to identify potential criteria. It also draws on the methods used to establish CONSORT (Consolidated Standards of Reporting Trials) (Moher, 1998) and the Cochrane collaboration’s tool for assessing risk of bias in trials (Higgins et al., 2011).

Therefore, at stage two, these criteria were put to a panel (Delphi) of senior researchers, who were asked to rate their appropriateness for assessing quality and human relevance in *in vitro* studies of brain tumours. Criteria reaching agreement form the basis of the checklist reported in this study.

### Summary of the Overall Approach

• *Pre-stage* (*documenting the need for a set of criteria/guidelines*)
  ∘ Search for and review of previously published systematic reviews in the field of *in vitro* cancer research.
  ∘ Examination of all papers described as systematic reviews of *in vitro* cancer studies to assess the quality/relevance assessment tools that had been used.

• *Stage one* (*identifying potential criteria*)
  ∘ Survey of senior brain tumour researchers to obtain a list of all *in vitro* techniques that are used in brain tumour research, suggested criteria, areas of agreement, relevant guidance, or quality-related initiatives.
  ∘ Interviews with a pre-defined sample of leading and emerging researchers, journal editors, senior clinicians, funding body, and regulatory committee representatives to elicit views on how quality and relevance should be assessed.
  ∘ Examination of peer review guidelines from major journals in the field for potential quality criteria for *in vitro* studies.
  ∘ Identification and analysis of all documents presenting potential quality criteria.
  ∘ Collation of the findings of the documentary analysis, and the survey and interview data.
  ∘ Development of a draft set of criteria for assessing *in vitro* studies based on the findings of the above.

• *Stage two* (*gaining agreement on criteria for the guidelines*)
  ∘ Establishment of a panel of experts in the field of brain tumour *in vitro* research.
  ∘ Use of the Delphi method to obtain agreement on key criteria to be used for assessing *in vitro* studies.

### Pre-stage

Searches were carried out using five databases for systematic reviews of *in vitro* cancer studies. All reviews that were described as a ‘systematic review’ and which focused solely on *in vitro* studies in any form of cancer were selected and the full-text checked for relevance. Those that met the inclusion criteria were selected and the data extracted on aspects including the focus and methods used. The full details of this review are to be published as a separate paper.

### Stage One

#### Quality Criteria in Previously Published Systematic Reviews

All relevant systematic reviews identified in the pre-stage review were selected and the full-text checked for mention of, or reference to, quality criteria, a checklist for quality, or guidance used to judge quality and/or human relevance of included studies.

#### Survey of Brain Tumour Researchers

An online survey was conducted to investigate current areas of research interest/focus related to *in vitro* research; *in vitro* models and study methods used within these areas; methods for assessing quality and relevance in these areas and knowledge of any published guidelines, checklists or quality initiatives.

Questions were developed by the authors with a draft version piloted followed by further revisions before the survey was finalised. The survey was completed online, using the University of Portsmouth’s online survey platform provider (Online Surveys.ac.uk) and was completed anonymously. A copy of the full set of questions in the questionnaire is available from the authors on request.

Potential participants were identified from conference abstracts for oral and poster presentations from international conferences and scientific meetings. See Table 1 for a list of sources used to identify potential participants. Once identified, further information was sought on participants from publicly available sources such as departmental, ResearchGate, and Google Scholar web pages. Those meeting inclusion criteria and for whom contact details could be found were approached for participation. Inclusion criteria were:

**TABLE 1:** Identification of potential participants by source.

Meeting/source	Abstracts screened (*n*)	Abstracts indicating *in vitro* research (*n*)	Potential participants excluded* (*n*)	Potential participants approached (*n*)
World Federation of Neuro-oncology Societies (WFNOS) 2017 Meeting (WFNOS, 2017a, b)	481	165	65	100
Society for Neuro-oncology (SNO) 2017 Annual Meeting (SNO, 2017a, b)	1248	224	56	168
European Association of Neuro-Oncology 13th Meeting (2018) (EANO, 2018)	446	157	61	96
British Neuro-Oncology Society (BNOS) 2017 Meeting (BNOS, 2018)	119	14	3	11
Asian Society for Neuro-Oncology (ASNO) 14th Meeting (2017) (ASNO, 2017)	240	52	27	25
Sub-Saharan Africa Neuro-Oncology Collaborative (S-SANOC) 2017 Planning Meeting (S-SANOC, 2017)	25	17	14	3
Additional (potential participants identified through other sources, e.g., team members or other neuro-oncologists)				33
Totals	2559	629	193	436

*Not in vitro specialist OR not neuro-oncology specialist OR no contact information available.

• Scientist working on studies of brain tumours using *in vitro* methods.
• Evidence of further publication history within brain tumour field using *in vitro* methods beyond conference abstract through which initially identified.

Potential participants were approached through published email addresses obtained from conference abstracts, departmental or professional web pages, or other publications. Design of invitations was informed by Fan and Yan’s (2010) recommendations drawn from a systematic review of factors affecting response rates in web surveys. Email invitations used a personalised salutation, identified survey tasks and salience, described how recipients were identified as potential participants, provided estimation of the time to finish the survey and gave contact details for further questions and assistance (Fan and Yan, 2010).

While no specific methodological guidance was available for conducting surveys of pre-clinical scientists, this target population was hypothesised to share many of the characteristics likely to affect participation which have been identified in previous studies with senior managers in other kinds of organisation. These include increased sensitivity to personalised responses, declining time capacity for participation due to increasing pressures from their core roles (Cycyota and Harrison, 2006), and an increasingly saturated information environment (e.g., email and social media) in which there are high levels of competition for feedback (and thus participant time). The potential vulnerability of the survey to low response in spite of efforts to implement best practise guidance formed part of the rationale for using multiple sources of data (i.e., survey, interviews, and documentary analysis) to inform the Delphi process, in order to make the project overall more resilient to the limitations of any single data collection stream.

#### Interviews With Key Individuals From the *in vitro* Field

Semi-structured interviews used widely to explore in-depth contextual factors affecting practise change (e.g., regulatory, funding, and variations in clinical or scientific practise) (Carlsen et al., 2007; Gardner and Webster, 2016; Colquhoun et al., 2017). A purposive sampling frame was constructed to reflect the different roles relevant to *in vitro* research. These included scientists, clinicians, regulators, and journal editors involved with studies using *in vitro* methods (including those working in fields other than brain tumour research, e.g., other neurological disease, other forms of cancer). Participants were identified through publications, professional and regulatory activities, and via the project team. An interview guide was developed and piloted with focus on the potential helpfulness and scope of set criteria, likely extent of agreement on assessment of *in vitro* research and the specific challenges in getting guidelines widely accepted.

The interviews aimed to explore:

• Professional opinion and practise in evaluation of quality and human relevance of *in vitro* models for brain tumour research, and identify points of agreement and disagreement.
• Current practises, opinions, and resources for identifying *in vitro* studies for review in brain tumour research.
• Factors that may promote or inhibit introduction of new practises for assessment of quality and human relevance in brain tumour research.

Interviews were carried out by phone after confirming consent with the participant. All interviews were guided by a set of core questions developed through pilot interviews, with follow up questions and exploration taking place where appropriate (and depending on the expertise and interest of respective participants). The semi-structured nature of interviews therefore meant that there was some variation in the length of interviews, number of questions asked, and in development of the interview schedule as the study progressed. Interviews were recorded and transcribed for directed content analysis in Nvivo (v12) Computer-assisted Qualitative Data Analysis Software (CAQDAS) (QSR International, 2018). Nvivo allows users to attach labels (or ‘codes’) to text, audio, video or image data, and facilitates data management through which directed content analysis can be conducted by a competent user. This involved reading across interview transcripts to identify responses relevant to the above aims (Corbin and Strauss, 2014).

#### Exploration of Author and Peer-Reviewer Guidance Provided by Journals

A set of relevant journals was identified using the following techniques:

• The 50 journals appearing most frequently in the results of searching the Medline database using the index term “*In vitro* Techniques+”.
• The 50 journals appearing most frequently using the search “*In vitro* Techniques+” AND “Neoplasms+” (both as index terms).
• The top 20 ranked journals from both the ‘Oncology’ and ‘Cancer Research’ categories of the Scimago Scientific Journal Rankings.

Resulting journals were combined into a single list, which after duplicate removal resulted in a set of unique journals for assessment of author and peer-review guidance. Assessment was conducted through manual exploration of journal websites, to identify publicly available information on author and peer-review guidance pertaining to quality assessment of *in vitro* methods.

#### Identification of Relevant Documents

Relevant documents including guidelines on the conduct and reporting of *in vitro* research, published standards were identified through the following methods:

• Previous review of published systematic reviews.
• 101 journal websites searched for general guidance on *in vitro*-relevant study reporting, and quality appraisal for specific techniques.
• Feedback from survey and interview responses.
• Searches of reporting guidance databases [e.g., US National Institutes of Health (NIH), EQUATOR Network FAIRSharing.org (2020)], the PubMed database and a commercial social networking site for scientists and researchers (ResearchGate).

All documents were loaded into NVivo software for directed content analysis.

#### Analysis

##### Survey

Descriptive statistical analysis was performed on quantitative data, and responses to free-text questions were analysed using a content analysis approach, where data area grouped into categories (e.g., cell line authentication and replication) for reporting (Krippendorff, 2018). For this aspect of the SAToRI-BTR project, data on quality and relevance criteria that are used in practise (e.g., when peer reviewing) and any quality initiatives or guidelines were extracted.

##### Interviews

Full interview transcripts were uploaded to Nvivo and directed content analysis of data was performed using Nvivo. Through this process, any data on quality and relevance criteria used in practise, and any quality initiatives or guidelines were identified and collated in order to inform stage two.

##### Documents

Directed content analysis was also performed on documents using Nvivo software.

#### Collation and Compilation of List of Proposed Criteria

A full list of all criteria was generated and a comparison of the criteria from documentary analysis compared with those from the survey and interviews. Any additional criteria generated from the latter were added to the list. The initial list was then further reviewed for duplicate criteria (i.e., those assessing the same or similar aspects but which were phrased in different terms which could be merged), ensuring that those criteria highlighted in several sources were retained and those that related only to a specialised technique were removed. The outcome of stage one was identification of a range of criteria for assessment of quality and/or human relevance in *in vitro* studies of brain tumours, which were organised in a taxonomy by area of focus.

### Stage Two

Having identified potential assessment criteria for *in vitro* studies of brain tumours at stage one through international survey, telephone interview, and documentary analysis, stage two involved evaluation of appropriateness of these criteria for assessment of brain tumour studies by a panel of senior scientists. The expert agreement panel (or ‘Delphi’) process has been used extensively in clinical and health services research to develop reporting guidance and quality assessment criteria for a range of scientific and clinical applications (Fitch et al., 2001; Boulkedid et al., 2011). Delphi allows participants to rate criteria anonymously (i.e., without knowledge of the composition of the panel, or identities of members), and to provide written feedback on them. The process occurs across multiple rounds, between which comments from all participants are also circulated so each participant is aware of the range of opinions and the reasons underlying these. Criteria reaching agreement are removed, additional criteria may be added, or existing criteria amended if they have not reached agreement (e.g., in response to suggestions from the panel). The process is anonymous and usually three rounds of the survey are sufficient to achieve reasonable agreement (Fitch et al., 2001; Hsu Chia and Brian, 2007; Boulkedid et al., 2011).

Delphi has been used both as a standalone technique to reach agreement on reporting and assessment criteria, and also as a sorting procedure to identify criteria of ongoing controversy requiring further discussion by a subsequent panel of experts (Boulkedid et al., 2011). As the aim of SAToRI-BTR is to identify agreed criteria, this method was assessed as appropriate for either outcome in terms of wider development of the project in the future.

#### Identification and Approach of Participants

Professors, heads of laboratories, and principal investigators who had been previously identified at either the survey or interview stages were invited to participate via personalised email and physical letter as described at the survey stage. Following indications of willingness to participate, participants were sent a link to complete the online consent form, after which they were invited to participate in the first round of the Delphi panel.

#### Development of Initial Criteria

Criteria identified at stage one were evaluated by expert members of the SAToRI-BTR Team and results grouped into categories for assessment by the panel.

#### Rating and Progression Between Rounds

**FIGURE 2:**
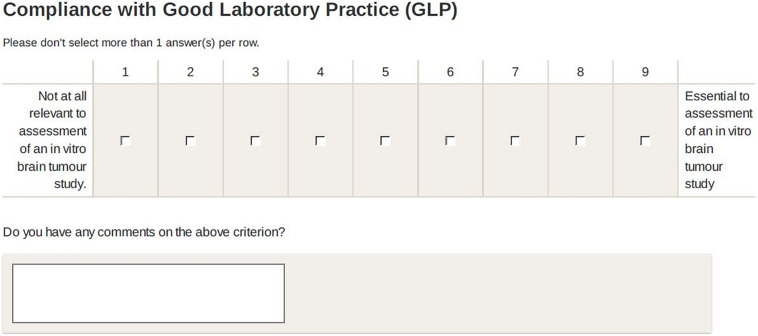
Example of Delphi rating scale and written feedback facility.

Participants rated criteria on an 9-point scale: 1 (not at all relevant) to 9 (essential) to assessment of a brain tumour study’. They were invited to leave qualitative comments (e.g., on context of application, clarity of criterion etc.) (Boulkedid et al., 2011) and to suggest additional criteria for each category (see Figure 2).

Criteria were judged to have reached agreement according to the RAND/UCLA agreement criteria (Fitch et al., 2001, p. 58). Criteria reaching agreement were removed between rounds, additional criteria added (if suggested by participants), and existing criteria not reaching agreement changed in line with participant feedback or (in its absence) passed to the next round unchanged.

The final outcome of stage two was a set of criteria around which agreement was obtained on their importance for the assessment of quality and relevance.

#### Ethical Approval

Ethical approval for the survey, interviews, and Delphi processes was granted by the University of Portsmouth Faculty of Science Ethics Committee, reference number SFEC 2018-073 (original application plus amendments).

## Results

### Pre-stage (Review of Published Systematic Reviews of *in vitro* Studies)

The review of published systematic reviews of *in vitro* studies indicated that few were available. Not all those described as systematic applied systematic approaches to the literature. Analysis of the methods used in the reviews confirmed that there was not a widely used set of criteria for assessing quality and/or relevance of *in vitro* studies. Those that did conduct a systematic appraisal of the included studies, adapted a wide range of existing appraisal checklists. A lack of agreed criteria specific to *in vitro* studies was highlighted.

### Stage One

#### Survey of *in vitro* Brain Tumour Researchers

**FIGURE 3:**
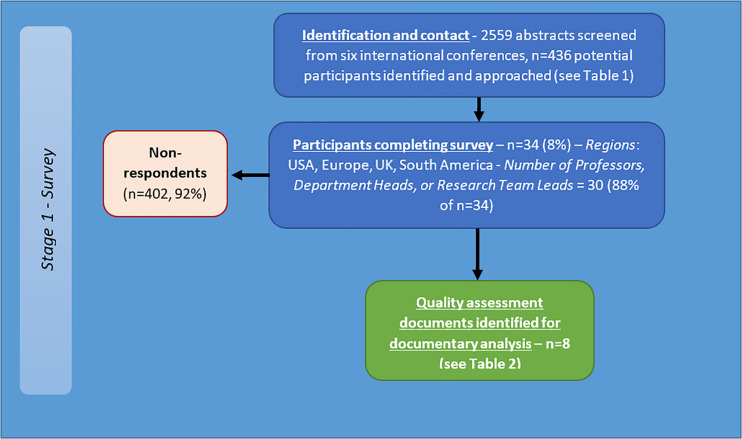
Flow diagram of recruitment and results for international survey of brain tumour researchers.

A total of 436 researchers were contacted and invited to complete the online questionnaire (see Figure 3). Of those invited, 7.8% (34 participants) completed the survey. Sixteen were from the United States, 12 from Europe, five from the United Kingdom and one from South America. A total of 10 different countries were represented. 30 (88%) participants identified as either ‘Professor/Department Head’ or ‘Research Team Lead.’ Mean years’ experience in brain tumour research 17.6 (*SD* = 10.1, range = 3–40). 28 participants (85%) also use *in vivo* techniques. The participants recommended a range of potential quality criteria.

#### Interviews

**FIGURE 4:**
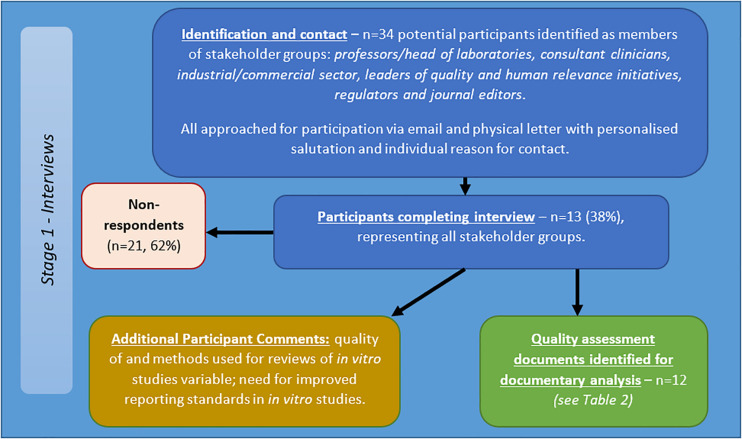
Flow diagram of recruitment and results for semi-structured interviews with stakeholders in *in vitro* studies of brain tumours.

Thirty-four potential participants approached via email and letter, of which 13 participants completed the interview (see Figure 4). The participants included/represented: professors/head of laboratories, consultant clinicians, industry, those leading quality and human relevance initiatives, regulators and journal editors. Telephone interviews totaled 414 min, with an average length of 34.46 min (range = 17–58 min, *SD* = 12.92 min).

Responses indicated:

• Methods for reviews of *in vitro* studies are highly variable;
• Quality of reviews of *in vitro* studies varies;
• Need for improved reporting standards.

Participants described a number of relevant quality assurance documents and reporting standards. Views were also expressed on the involvement of clinicians and regulators in development and barriers to adoption of any suggested guidelines or quality initiatives. More detailed results are to be reported in subsequent published works.

#### Exploration of Author and Peer-Reviewer Guidance Provided by Journals

Of the 101 unique journal titles identified, 6 journals had been discontinued or renamed, 2 were book series and 1 journal was inaccessible. Identifying and including the replacement titles for the renamed journals resulted in a total of 96 journals currently in print and accessible which could be assessed. Fifty-eight (60%) journals did provide some guidance specific to or relevant to *in vitro* techniques. Thirty-eight (40%) did not appear to provide any guidance accessible on the website that was specific to *in vitro* research. Established guidelines were reported by 25 journals (26%). These guidelines included those on specific techniques which could be, but are not exclusively, applied in *in vitro* research, such as MIAME (Minimum Information About a Microarray Experiment) and STRENDA (Standards for Reporting Enzymology Data). Generic guidelines were also cited such as the National Institute for Health (NIH) Principles and Guidelines for Reporting Preclinical Research. Cell line authentication was referred to by 22 journals and related guidance that was cited included the UKCCCR Guidelines for the Use of Cell Lines in Cancer Research. A full list of the guidance that was located through review of the journal websites is included in Table 2.

**TABLE 2:** Quality assessment documents identified for analysis including sources.

Documents	Survey	Interviews	Searches of journal criteria and assessment databases
Tools for methodological quality and risk of bias (Al Saadi et al., 2016)			Y
Cell culture techniques [edited collection] (Aschner et al., 2011)			Y
Minimum information about a microarray experiment (MIAME)—toward standards for microarray data (Brazma et al., 2001)			Y
STAR methods guide (CELL Press, 2020)	Y		Y
Six checklists relating to *in vitro* models for different organs/systems [COMMERCIAL ENTERPRISE]*		Y	
Quality of reporting in systematic reviews – meta-analyses of *in vitro* studies – a systematic review protocol (Elshafay et al., 2019)			Y
Good cell culture practises and *in vitro* toxicology (Eskes et al., 2017)			Y
EU-NETVAL meeting 10–11th October 2016 (EU-NETVAL, 2016)		Y	
EU-NETVAL meeting 26th–27th November 2015 (EU-NETVAL, 2015)		Y	
GOOD *IN VITRO* METHOD PRACTISES (GIVIMP) (OECD, 2018)		Y	
EURL ECVAM workshop – inaugural meeting of EU-NETVAL members – 26–27 June 2014 (EURL ECVAM, 2014)		Y	
Guidelines for the use of cell lines in biomedical research (Geraghty et al., 2014)			Y
Perspectives on *in vitro* to *in vivo* extrapolations (Hartung, 2018)		Y	
Hartung et al. (2002) good cell culture practise ECVAM good cell culture practise task force report 1 (Hartung et al., 2002)		Y	
Definitions relating to cell line authentication (ICLAC, 2019b)	Y	Y	Y
Cell line checklist for manuscripts and grant applications (ICLAC, 2019a)	Y	Y	Y
Better reporting for better research: a checklist for reproducibility (Kenall et al., 2015)	Y	Y	Y
UKCCCR guidelines for the use of cell lines in cancer research (UKCCCR, 2000)			Y
Reporting recommendations for tumour marker prognostic studies (REMARK) (McShane et al., 2005; Altman et al., 2012)	Y	Y	Y
Enhancing reproducibility through rigour and transparency (NOT-OD-15-103) (NIH-OER, 2015)	Y		Y
Guidelines for research involving recombinant or synthetic nucleic acid molecules (NIH, 2019)	Y		Y
Principles and guidelines for reporting preclinical research (NIH, 2017)	Y		Y
Advisory document of the working group on good laboratory practise the application of the principles of GLP to *in vitro* studies (OECD, 2004)		Y	
Good cell culture practise for stem cells and stem-cell-derived models (Pamies et al., 2017)			Y
Extending a risk-of-bias approach to address *in vitro* studies – a systematic review protocol (Rooney, 2015)			Y
*In vitro* acute and developmental neurotoxicity screening – an overview of cellular platforms and high-throughput technical possibilities (Schmidt et al., 2017)			Y
Promoting coherent minimum reporting guidelines for biological and biomedical investigations – the MIBBI project (Taylor et al., 2008)			Y

*Documents marked [COMMERCIAL ENTERPRISE] were provided on condition of confidentiality of content.

For all journals still in publication and with an impact factor for 2018 (*n* = 95), the median impact factor (IF) was 4.9 (range = 0.6–223.7, IQR = 5.6). Of these: for journals citing established criteria (*n* = 25) the median was 5.2 (range = 1.9–59.1, IQR = 3.5); for journals giving general guidance (*n* = 48) the median was 4.9 (range = 1.9–41.1, IQR = 3.5); while for journals giving no specific guidance (*n* = 37) the median was 4.5 (range = 0.6–223.7, IQR = 6.7).

The analysis of author and peer-review guidance provided further evidence to support the observation of a lack of common, comprehensive, quality assessment criteria for *in vitro* studies, by showing significant variation in the quantity and types of guidance provided by journals.

#### Documentary Analysis

**FIGURE 5:**
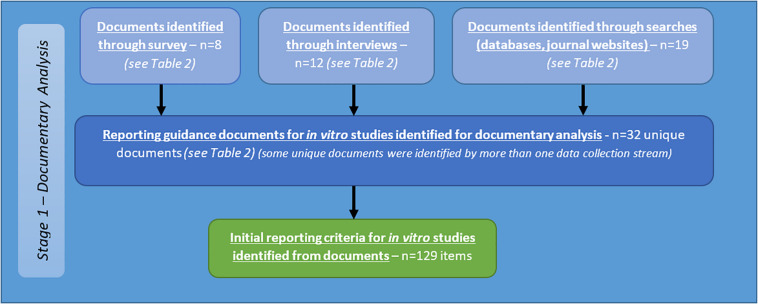
Flow diagram describing sources of identification of reporting guidance documents, and initial criteria for reporting of *in vitro* studies of brain tumours resulting from documentary analysis.

A total of 32 documents were identified from the above sources (see Table 2). Criteria identified from analysis of these documents are reported below as part of the summary of all criteria identified from stage one (see Figure 5).

### Stage Two

#### Compilation of Preliminary Criteria

**FIGURE 6:**
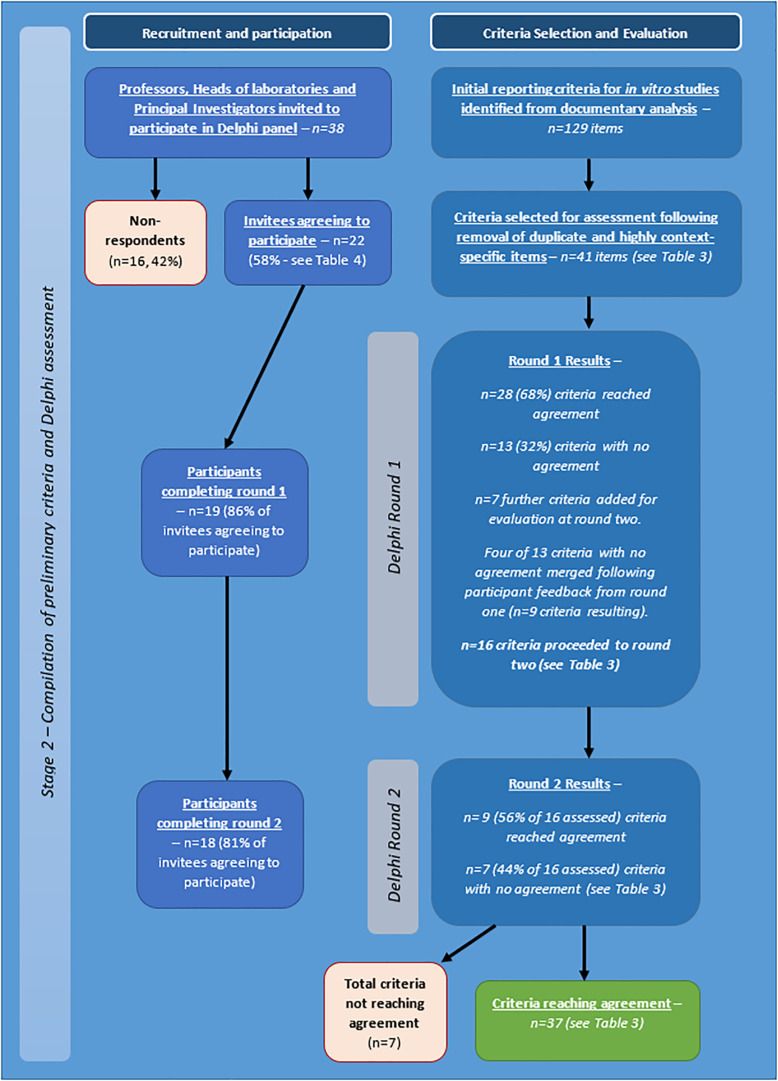
Flow diagram describing Delphi process and outcomes.

Following completion of the survey, interview, and documentary analysis stages, potential criteria were collated using Nvivo software, through which a long list of 129 preliminary items were identified (see Figure 6). Duplicate and highly context specific items were removed. Forty-one criteria were selected for assessment by expert panel (Delphi) (see Table 3).

**TABLE 3:** Summary of Delphi process (italics: no agreement).

Category	Criteria Delphi round 1	Agreement (*n* = 19)	Median	Criteria Delphi round 2	Agreement (*n* = 18)	Median	Criteria reaching agreement
General	*Ethical approval*	*N*	*5*	*Changed to: Ethical approval for cells from human donors*	*N*	*8*	–
	Compliance with Good Laboratory Practise (GLP)	Y+	9		–		Compliance with Good Laboratory Practise (GLP)
Initial set-up and processes	Transportation conditions for tissues/cells	Y+	9		–		Transportation conditions for tissues/cells
	*Quarantine process for new cells*	*N*	*8*	Changed to: Quarantine process in place for cells introduced from other laboratories	Y+	9	Quarantine process in place for cells introduced from other laboratories
	Testing for micro-organisms	Y+	9		–		Testing for micro-organisms
	Cell authentication	Y+	9		–		Cell authentication
	Method of primary culture establishment	Y+	9		–		Method of primary culture establishment
	*Cell detachment and disaggregation methods*	*N*	*7*	Unchanged	Y+	7	Cell detachment and disaggregation methods
	*Take rate of primary culture establishment*	*N*	*6.5*	*Changed to: Success rate for establishing primary culture*	*N*	*8*	–
	Sources of reagents	Y+	9		–		Sources of reagents
	Consistent use of reagents	Y+	9		–		Consistent use of reagents
Cells	Origin or source of cells (whether tissue, biopsy-derived early passage or cell lines)	Y+	9		–		Origin or source of cells (whether tissue, biopsy-derived early passage or cell lines)
	Cell authenticity	Y+	9		–		Cell authenticity
	*Genomic stability*	*N*	*7*	*Changed to:* Researcher awareness of genomic instability	Y+	9	Researcher awareness of genomic instability
	Passage number (reduced heterogeneity and acquired resistance)	Y+	9				Passage number (reduced heterogeneity and acquired resistance)
	Cell characterisation (morphology, differentiation and antigenicity)	Y+	9				Cell characterisation (morphology, differentiation and antigenicity)
	*Population doubling times*	*N*	*8*	Unchanged	Y+	7	Population doubling times
	Cell viability testing	Y+	8		–		Cell viability testing
	Cryopreservation process/method	Y+	9		–		Cryopreservation process/method
Models	Patient-derived (human)	Y+	9		–		Patient-derived (human)
	Cellular heterogeneity	Y+	8		–		Cellular heterogeneity
	Culture conditions 1 (HEPES or CO_2_ incubation)	Y+	9		–		Culture conditions 1 (HEPES or CO_2_ incubation)
	Culture conditions 2 (temperature, oxygen, pH, and humidity)	Y+	9		–		Culture conditions 2 (temperature, oxygen, pH, and humidity)
	Serum supplementation (human, FCS/NCS or serum-free)	Y+	9		–		Serum supplementation (human, FCS/NCS, or serum-free)
	Complexity of the model (3D versus 2D)	Y+	9		–		Complexity of the model (3D versus 2D)
	*Vascular flow*	*N*	*5*	Merged *	–		–
	*Use of antimycotics and/or antibiotics*	*N*	*7*	Changed to: If used, effect of antimycotics and/or antibiotics on cell growth	Y+	7	If used, effect of antimycotics and/or antibiotics on cell growth
	*Tumour microenvironment (e.g., immune cells and non-neoplastic glial cells)*	*N*	*8*	*Unchanged*	*N*	*9*	–
	*Representation in the model of the physiology of intended patients*	*N*	*7*	Merged *	–		–
Assays	*Multimodality assays*	*N*	*7*	Changed to: Validation of results using multiple methods	Y+	7.5	Validation of results using multiple methods
	Functional assays (biological behaviour)	Y+	8		–		Functional assays (biological behaviour)
	Replicated assays	Y+	9		–		Replicated assays
	Appropriate controls	Y+	9		–		Appropriate controls
	*Therapeutic testing*	*N*	*7*	Merged **	–		–
	*Blood brain barrier*	*N*	*6*	Merged *	–		–
Interpretation by authors	Reproducibility of results (within-laboratory)	Y+	9		–		Reproducibility of results (within-laboratory)
	Reproducibility of results (between laboratory transferability)	Y+	8		–		Reproducibility of results (between laboratory transferability)
	Definition of the human relevance of the *in vitro* model	Y+	8		–		Definition of the human relevance of the *in vitro* model
	Demonstration of the relationship of the model to the target tissue or organ	Y+	9		–		Demonstration of the relationship of the model to the target tissue or organ
	Discussion of the limitations of the method	Y+	9		–		Discussion of the limitations of the method
	Discussion of the limitations of the model	Y+	9		–		Discussion of the limitations of the model
Additional criteria proposed by Delphi panel members		–	–	Reporting of Standard Operating Procedures (SOPs) to aid replication	Y+	9	Reporting of Standard Operating Procedures (SOPs) to aid replication
		–	–	Pathology/patient data reported or accessible	Y+	9	Pathology/patient data reported or accessible
		–	–	*Sampling of different regions of heterogeneous tumour*	*N*	*7*	–
		–	–	*Time interval between collecting biopsy tissue and setting up primary culture*	*N*	*9*	–
		–	–	Substrate on which cells are cultured	Y+	8.5	Substrate on which cells are cultured
		–	–	*Assessment of imaging method used*	*N*	*7*	–
		–	–	*Appropriate use of bioinformatics and/or mathematical modelling*	*N*	*7*	–

* Criterium merged with Demonstration of the relationship of the model to the target tissue or organ (Criteria Delphi round 1). ** Criterium merged with Multimodality assays (Criteria Delphi round 1).

#### Expert Panel

Of the 38 professors, heads of laboratories, and principal investigators invited to participate by personalised email and letter, 22 agreed to participate initially. 19 participants completed round one and 18 completed round two (see Figure 6). For a comparison of those participating in the expert panel compared with those taking part in the survey and interviews, see Table 4.

**TABLE 4:** Comparison of participants in survey, interviews and Delphi.

Country	Surveys	Interviews	Delphi	Minimum number of individuals participating in at least one stage	
				Brain tumour *in vitro* researchers	All participants
Belgium	1		1	1	1
Brazil	1			1	1
Germany	4	1	2	4	5
Ireland		1	2	2	2
Italy	1		1	1	1
Luxembourg	1			1	1
Netherlands	3			3	3
Norway	1		2	2	2
Poland			1	1	1
Slovenia			1	1	1
Sweden	1			1	1
United Kingdom	5	11	7	7	15
United States	16		2	16	16
Total	**34**	**13**	**19**	**41**	**50**

#### Delphi Assessment

Of the initial 41 criteria, agreement was achieved in round one on 28 with no agreement on 13. Seven further criteria were suggested.

For round two, based on feedback from participants, four of the 13 criteria were merged with existing criteria, nine were re-presented to Delphi group (with or without rephrasing) and the seven new criteria identified by participants were also presented to the panel. Thus, a total of 16 criteria were assessed in round two and a total of 48 criteria across the two rounds.

In round two agreement was achieved on nine (including three new criteria). No agreement was apparent on seven criteria (including four new criteria). Although the level of agreement across the two rounds of Delphi completed was high, for the remaining seven criteria, the level of agreement, even when criteria were rephrased reduced and the feedback from participants indicated that achieving agreement on these was unlikely to be feasible.

The large amount of qualitative data to analyse (comments, explanations etc.) collected should further inform the application of the criteria in practise. Further specification of criteria needs discussion and, thus, it was decided that the next stage would require an in-person meeting and discussion. A summary of the process is shown in Table 4, and in Figure 6.

## Discussion

This study represents a first attempt to use a systematic approach to generating a set of criteria for assessing the quality and relevance of *in vitro* brain tumour research studies. In designing the process, the aim was to combine a systematic analysis of existing guidance and practise with the implicit views of those with expertise in *in vitro* brain tumour research. By attempting to engage those involved in research in this field at an early stage and throughout the process, it was anticipated that the uptake of any resulting guidance would be optimised. It was also anticipated that focusing the study in a specific area (brain tumour research) would also increase the relevance and hence engagement with the process.

There have been a number of initiatives aimed at improving and standardising the quality and reporting of *in vitro* research, some of which are ongoing (Eskes et al., 2017; Pamies et al., 2017; OECD, 2018; Hartung et al., 2019). Full and transparent reporting is of importance as it enables the evaluation and reproduction by other researchers and thus optimises the resources that have been expended. While various initiatives have been undertaken, adoption by researchers working in the field has been low (Pamies et al., 2017). The participants taking part in the survey and interviews conducted in this study were aware of some of these initiatives but there was not universal or consistent reference to any particular set of guidance.

The intention of screening a large number of abstracts from a series of relevant recent conferences was to ensure that a large number of researchers were involved in the survey (and thus the guideline production process). Over 2,500 abstracts were screened resulting in over 400 individual senior researchers being identified but the response rate was extremely low. There may be several explanations for this: lack of perceived relevance or concern about the ultimate aim of any guidance may have discouraged participation. Other practical problems such as contact emails being filtered out by organisational email servers may also have had an impact. Additionally, the field of brain tumour research has, historically, been poorly funded (House of Commons Petitions Committee [HCPC], 2016) so that researchers are likely to be focused on core issues including grant income and job security and we anticipated that they may be less inclined to become involved in research that appears more peripheral to these aspects. Because of this, we chose a study design (as summarised in Figure 1) which drew on multiple data collection streams to inform the criteria for Delphi assessment (survey, interview, and documentary analysis). This was to ensure that the study design was resistant to risks associated with low response rates to survey or interviews. Nevertheless, more than 30 heads of laboratories/professors from 10 different countries did participate and, overall 40 *in vitro* brain tumour experts from 13 countries contributed to at least one stage of the process. This is a significant number in a relatively small field and these people represent experienced, senior authorities within the field. Furthermore, there was consistency in the criteria suggested in the survey, interview, and documents.

There was also consistency and a high level of agreement on the importance of each of the criteria proposed. The final outcome of the process reported in this paper is a set of 37 criteria which reached agreement as essential to consider when assessing the quality and/or human relevance of an *in vitro* study. The focus was on *in vitro* research in the brain tumour field but the majority of the criteria generated are generic and could be applied to other *in vitro* research areas, particularly those in the field of cancer.

The data collected included individual comments and feedback on each of the criteria and revealed areas where there are differences in opinions and practise which would benefit from further investigation. It will be necessary to further specify how each of the ‘criteria’ could best be applied in practise as, in some cases, this is implicit and/or context-specific rather than explicit and/or universal. Thus, criteria refer to specific aspects of an *in vitro* study that should be assessed and not whether simply reporting this would constitute good practise or whether it is also necessary that the study meets a particular standard related to this aspect. For example, there was agreement on the importance of assessing ‘cellular heterogeneity’ but no specific standard of reporting or conduct is currently attached to this. The data collected will inform the next stage of the process which is to develop more detailed guidance on the application of the criteria in practise. This may require an in person meeting as is generally required for finalising guidance such as this (EQUATOR Network, 2020). Ultimately, the set of guidance generated could be disseminated and used by journals, grant awarding bodies, and peer reviewers. As has been proposed, producing and disseminating a set of agreed criteria for the assessment of *in vitro* studies will further support ‘Meaningful contributions to the body of science’ as they can evaluated and reproduced by other researchers in the field and are more accessible to those in related fields previously (Hartung et al., 2019).

## Conclusion

The SAToRI-BTR project drew on a range of well-established methods for identification and appraisal of current practise standards. Through a rigorous, systematic process of expert review, the project has resulted in a set of preliminary criteria for use in assessment of quality and human relevance of *in vitro* brain tumour studies. Further development of these criteria, including potential strategies for adaptation and dissemination across different sub-fields of brain tumour research, will follow. While the focus of the study remains in the brain tumour field, the initial criteria identified and the methods through which they were developed remain applicable to a broader range of fields relating to *in vitro* research. It is therefore hoped that this investigation will prove useful empirically and methodologically, both within and beyond the specific focus of brain tumour studies.

## Data Availability Statement

The datasets presented in this article are not readily available because the small numbers of participants and specific nature of the responses mean that individuals may be identified. Anonymised data collated from the online survey are available on request from the authors. Requests to access the datasets should be directed to KP, karen.pilkington@port.ac.uk.

## Ethics Statement

The studies involving human participants were reviewed and approved by University of Portsmouth Faculty of Science Ethics Committee, reference number SFEC 2018-073 (original application plus amendments). The patients/participants provided their written informed consent to participate in this study.

## Author Contributions

KP and GP conceptualised the study. MB, GP, CH, and KP contributed to the design of the study. MB conducted the data collection. MB and KP carried out the analysis. MB and KP drafted the manuscript. All authors contributed to the final version of the manuscript.

## Conflict of Interest

The authors declare that the research was conducted in the absence of any commercial or financial relationships that could be construed as a potential conflict of interest.
